# Effects of a competitive season on the plasma lipid profile of soccer players

**DOI:** 10.14814/phy2.70382

**Published:** 2025-05-27

**Authors:** Santo Marsigliante, Giulia My, Okba Selmi, Enrico Paolo Villani, Aymen Hawani, Antonella Muscella

**Affiliations:** ^1^ Department of Biological and Environmental Sciences and Technologies (Di.S.Te.B.A.) University of Salento Lecce Italy; ^2^ High Institute of Sports and Physical Education of Kef, University of Jendouba Kef Jendouba Tunisia; ^3^ Physical Activity, Sport and Health, Research Unit (UR18JS01) National Observatory of Sport Tunis Tunisia

**Keywords:** cholesterol, coronary heart disease, high‐density lipoprotein, low‐density lipoprotein, soccer, triglyceride

## Abstract

This study aimed to evaluate the impact of a soccer season on the lipid profile of professional soccer players. Forty male players participated in the study. Blood samples for lipid profile analysis were collected at four time points: before the start of the training period (T0), immediately after the 4‐week training phase (T1), at mid‐season (T2, 23 weeks after T0), and the end of the season (T3, 43 weeks after T0). Throughout the competitive season, there was a significant decrease in body fat percentage (BFP, *p* < 0.001, RM‐ANOVA) and an increase in fat‐free mass (FFM, *p* < 0.001, RM‐ANOVA). Plasma levels of total cholesterol (TC) and LDL cholesterol (LDL) significantly decreased (TC: *p* = 0.003, LDL: *p* = 0.033, RM‐ANOVA), whereas HDL cholesterol (HDL) levels increased significantly in response to training (*p* = 0.013, RM‐ANOVA). Triglyceride (TG) concentrations were significantly higher before the season than after (*p* < 0.001, RM‐ANOVA). Additionally, the LDL/HDL and TC/HDL ratios showed significant reductions over the season (LDL/HDL: *p* = 0.004, TC/HDL: *p* < 0.001, RM‐ANOVA). The observed decrease in LDL, TC, and TG levels, along with the increase in HDL levels at the end of the season. These results suggest that regular soccer training induces favorable changes in the lipid profile of professional players. Further studies are needed to determine whether such changes are associated with long‐term health outcomes in elite athletes, particularly considering their typically low baseline risk for cardiovascular conditions.

## 1 INTRODUCTION

Physical activity is associated with decreased cardiovascular morbidity and mortality (Kraus et al., 2002; Kraus et al., 2018).

Measuring plasma lipids—specifically plasma LDL‐cholesterol (LDL) concentrations—is the most universal approach to evaluating lipoprotein biomarkers to assess risk for atherosclerosis and coronary heart disease (CHD). However, as with many biomarkers, there are limitations and exceptions to plasma lipids‐CHD associations since plasma lipid measures cannot comprehensively depict the highly dynamic and systemic processes involved in lipoprotein metabolism and cellular interactions (Andersen & Fernandez, 2025). Accordingly, it has been known for some time that individuals with normal LDL levels have shown occlusion in the coronaries or have even experienced myocardial infarctions (Hua & Malinski, 2019). Since small, dense LDL particles are more susceptible to oxidation and can be the beginning of plaque accumulation, it has now helped acknowledge that the size of the LDL particle is an important factor to consider for diagnosing (CHD) risk. This knowledge strongly suggests that measuring plasma LDL‐C alone does not provide a complete picture of (CHD) risk (Muscella, Stefàno, & Marsigliante, 2020).

Then, risk factors for CHD are low high‐density lipoprotein (HDL) levels and high triglycerides (TGs), cholesterol, and blood lipids concentrations (Carey et al., 2010). In addition, a high body mass index is associated with unfavorable lipoprotein profiles, with an increased risk of CHD and mortality (Shamai et al., 2011). HDL effects mirror its cholesterol efflux activity and its antioxidant, antidiabetic, anti‐inflammatory, and antithrombotic properties (Franczyk et al., 2023).

Consistently, low HDL levels are usually accompanied by high triglyceride (TG) levels, insulin resistance, and abdominal obesity (Wing & Greeno, 1994). HDLs are of different sizes and lipid compositions. Both the quantity and quality of HDL appear to be influenced by genetic factors, aging, disease states, exposure to some environmental factors and pathogens, smoking, and diet (Cho, 2022). Furthermore, HDL status also depends upon lifestyle, such as physical activity (Muscella, Stefàno, Lunetti, et al., 2020), and a sedentary lifestyle is related to low HDL levels, irrespective of age and sex (Julian et al., 2022; Li & Chen, 2021). It follows that, in primary prevention, physical activity may well be a way to control this CHD risk factor, also having a beneficial effect on the serum lipid profile. It is known that both aerobic and anaerobic exercise modulates the lipid structure by increasing HDL (Szapary et al., 2003; Wang & Xu, 2017) and decreasing TG; exercise effects on LDL generally are regarded to be minor in magnitude and associated with an improvement in the size of these lipoprotein particles (Steinmetz et al., 2001). Obviously, the response of the lipid profile to an exercise session or training program is different depending on the type of exercise undertaken, its intensity and frequency, duration of each session, and time spent in such a program (Sousa et al., 2014). Furthermore, there is a wide variability in the HDL‐raising effect of physical activity, possibly due to basic environmental and genetic differences (Muscella, Stefàno, & Marsigliante, 2020; Muscella, Stefàno, Lunetti, et al., 2020). Thus, thresholds for an exercise effect on lipids and lipoproteins are hard to identify (Boraita, 2004). Nonetheless, it is quite clear that high cholesterol and low HDL‐C levels are more present in power sports (i.e., boxing, wrestling, weightlifting, and judo) and anaerobic sports (i.e., sprints and jumps, tennis, ice skating, and gymnastics) (Mann et al., 2014).

Fat and carbohydrate are the main energy substrates used during exercise; their respective intake is largely regulated by exercise intensity, although other factors such as exercise duration, age, gender, diet, physical condition, and substrate availability are also involved. Fatty acids are the most abundant source of endogenous energy substrates during exercise at intensities between 45% and 65% of VO_2_max (Muscella, Stefàno, Lunetti, et al., 2020). At the beginning of low‐intensity exercise, lipolysis increases further, thus providing sufficient FFA to provide energy substrates over requirements. However, lipolysis does not increase further with increasing exercise intensity, and fatty acid oxidation becomes approximately equal to the total amount of available fatty acids at 65% of VO_2_max. During high‐intensity exercise (>80% of VO_2_max), however, carbohydrate becomes the major substrate (Wolfe, 1998). The combination of aerobic and resistance training may be the optimal combination to improve serum lipoproteins, but further research is needed on the safety and efficacy of this combined approach, focusing on issues of frequency and duration.

For this reason, recently there has been interest in biology and the effects of soccer on physiological and biochemical factors, especially lipid profiles (Sotiropoulos et al., 2008). Soccer is the most popular sporting event in the world, and from the point of view of physical activity, it is an intermittent sport, characterized by acyclic and unpredictable changes of activity, including sprints, changes of direction, tackles, and jumps. Soccer players had significantly lower serum cholesterol/HDL ratio (by 15%) and higher HDL (by 18%) than nonathletes (Amanatidis et al., 2006). In addition, HDL levels further increased significantly after the match (Sotiropoulos et al., 2008). Also, there was a significant decrease in TGs and total cholesterol (Sotiropoulos et al., 2008). This data suggests that intermittent exercise of long duration, such as a soccer match, will result in an acute antiatherogenic modification of lipid profile, possibly due to the high aerobic energy expenditure. However, only limited research has studied the effects of a soccer match on serum lipid profiles in professional players (Amanatidis et al., 2006; Garry & McShane, 2001), and no study has explored intense training over a long period.

This study investigates the impact of intensive in‐season training on the blood lipid profile of elite male soccer players. By examining the effects of sustained high‐intensity training across different phases of the competitive season, we aim to better understand how such training influences blood lipid markers.

These insights could contribute to a deeper understanding of exercise‐induced changes in blood lipid profiles in high‐performance athletes, with potential implications for how similar training patterns might benefit broader populations as well.

## 2 MATERIALS AND METHODS

### 2.1 Experimental design

**FIGURE 1:**
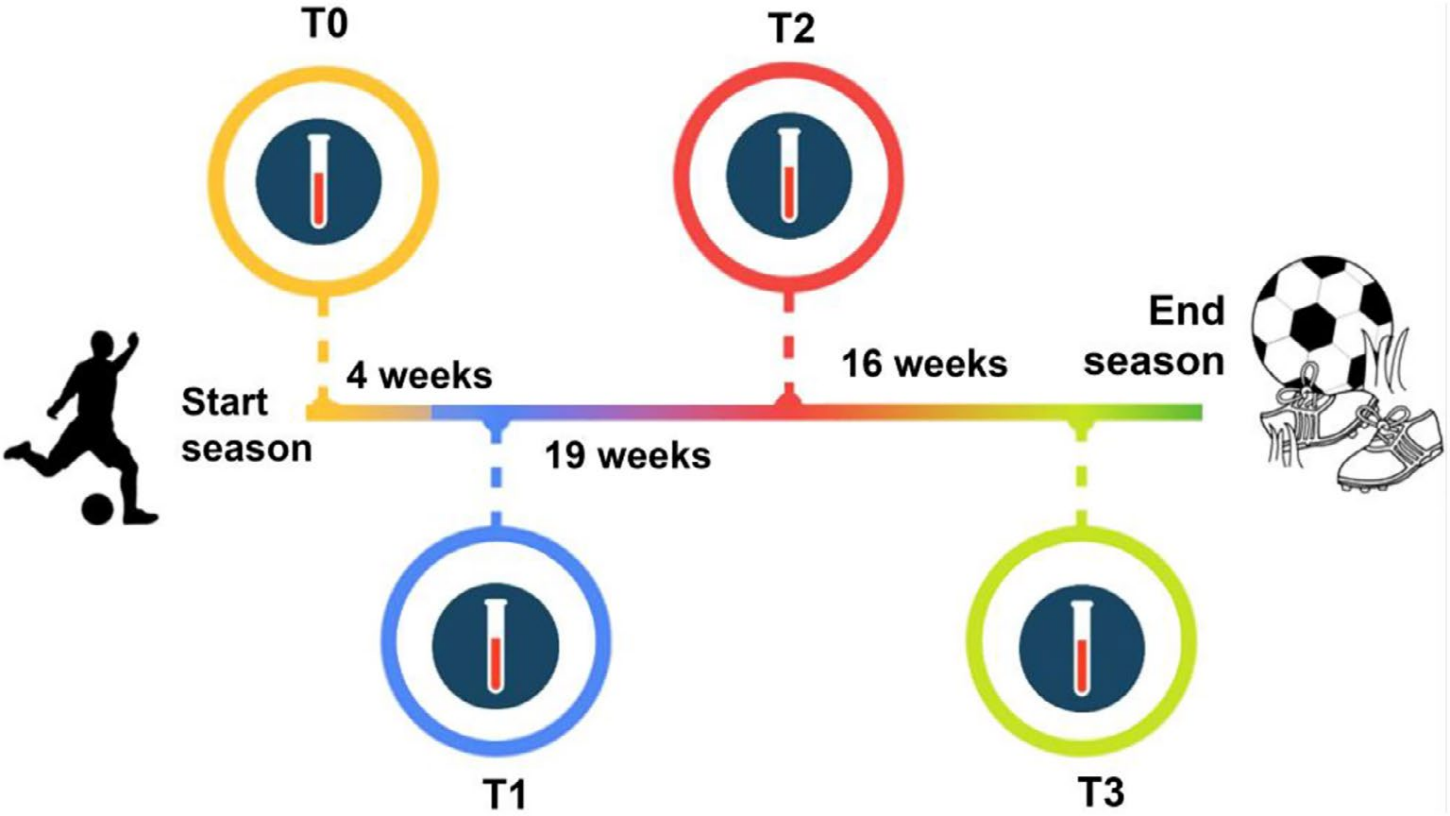
Timeline of season.

Male soccer players were evaluated at four different time points (Figure 1): before the beginning of the training period (T0), just after the 4‐week training period (T1), at the middle of the season (T2, after 23 weeks from T0), and the end of the season (T3, after 43 weeks from T0). The blood samples were collected 24 h after different matches, at 7.30 a.m., in the fasting state. Physical tests and anthropometric measurements were performed in each period (T0, T1, T2, T3) to assess the athletes physical condition.

### 2.2 Subjects

Based on an a priori calculation conducted using G*Power 3.1.9.4 software (version 3.1, University of Düsseldorf, Düsseldorf, Germany), it was determined that a sample size of 36 participants would be required to detect a medium effect size of *d* = 0.82 with 95% power and an alpha level of 0.05.

Forty soccer players (range 16–31 years; mean ± SD: age, 22.6  ± 4.6 ) competing in the highest Italian league voluntarily took part in this study.

Only athletes with stable medical conditions were included. The study excluded athletes with medical conditions confirmed by their medical history, including severe musculoskeletal disorders (such as recent fractures or severe osteoarthritis), uncontrolled endocrine disorders (such as hypothyroidism or hyperthyroidism), significant psychological conditions (such as severe depression or anxiety disorders), and infectious diseases or conditions requiring prolonged periods of incapacity. Additionally, injured athletes and those with less than 1 year of soccer training experience were excluded. Athletes on medications affecting metabolism or lipid profiles were excluded. Relevant medication use was identified during screening, and athletes on treatments that could interfere with study outcomes were not enrolled.

The study was carried out according to the Declaration of Helsinki. All participants read and signed an informed consent form approved by the Institutional Review Board (I.R.B.) of the Department of Biological and Environmental Science and Technologies (Di.S.Te.B.A.) (protocol code N‐1/2021 and 7 January 2021).

### 2.3 Soccer training

Table 1 shows the training program followed by the team both during the first 4 weeks of the athletic preparation phase (Pre‐season) and during the competitive period of the season, 39 weeks long.

**TABLE 1:** Team training program, divided into season and period of athletic preparation (pre‐season).

Category	Preseason	In‐season
Training focus	Physical development and general conditioning	Performance maintenance and match readiness
Aerobic training (%)	45%	30%–35%
Anaerobic training (%)	15%–20%	20%–25%
Other training (%)	35%–40% (mobility, core, technique, etc.)	40%–45% (tactics, recovery, gym)
Typical weekly activities	Week 1: Gradual return to training with light aerobic work, core strength, proprioception, and individual technique. Week 2: Intermittent endurance development, general muscle strengthening, and technical and tactical workshops. Week 3: Intensification with HIIT, competitive short‐form games, speed work, and a first friendly match. Week 4: Volume reduction, maintaining intensity, tactical sessions, and a full friendly match.	2–3 high‐intensity technical‐tactical sessions 1–2 low‐intensity technical‐tactical sessions 1–2 strength or speed sessions (gym) 1–2 matches per week
Training sessions × week (*n*)	8–10 (with double sessions)	5–7
Average daily training duration	90 min (range: 60–120 min)	60–90 min
Average weekly training load (AU)	2500–3500 AU (higher cumulative workload)	1500–2500 AU (adjusted for match recovery)

*Note*: Please refer to the Tables S1 and S2 for the detailed weekly training tables for preseason and season, which include the quantification of volume and training load.

Abbreviations: AU, (sRPE×min); Km, total distance.

Training load (expressed in Arbitrary Units, AU = session RPE × duration) was calculated using the session‐RPE method, obtained by multiplying the perceived exertion (RPE, Selmi et al., 2022) by the session duration in minutes. External load parameters, including total distance covered (in kilometers), were recorded using a GPS tracking system (e.g., 10 Hz GPS units, STATSports), worn by each player during training sessions and matches. All measurements were conducted by the same trained and experienced researcher throughout each phase of the study to ensure methodological consistency and minimize the risk of measurement bias.

We did not perform a detailed quantitative assessment of physical activity levels during the offseason, which is typically characterized by a significant reduction in training intensity and volume. During this period, players generally maintain only low to moderate levels of activity, often self‐directed and unstructured, compared to the rigorous demands of in‐season training and matches. This reduction in baseline physical activity before T0 may have contributed to the subsequent improvements observed during the season.

All training sessions were preceded by a standardized warm‐up of 5–15 min.

### 2.4 Diet

Table 2 presents estimated dietary intake for the ‘Pre‐Season’ and ‘Season’ phases, based on the average intake calculated using dedicated nutritional analysis software (ESHA Food Processor SQL Nutrition and Fitness Software version 10.5). These estimates were developed by the club's medical team with the assistance of a nutritionist, following current sports nutrition guidelines (Collins et al., 2021; Danielik et al., 2022; Książek et al., 2020). The ‘Pre‐Season’ phase corresponds to the period from T0 to T1, while the ‘Season’ phase spans from T1 to T3, with dietary monitoring conducted regularly throughout the competitive cycle. Athletes were encouraged to follow these diets and to consume fluids regularly throughout the day, especially before, during, and after training sessions or matches. All participants were provided with the same supplements throughout the study, including multivitamins and electrolytes. These were carefully selected to ensure adequate micronutrient intake and support hydration without affecting substrate utilization or metabolic function, minimizing potential impact on the study's primary outcomes.

**TABLE 2:** Food and dietary intake.

Nutrient	Pre‐season (mean ± SD)	Season (mean ± SD)
Carbohydrates (g/day)	406.2 ± 48.1	332.8 ± 102
Proteins (g/day)	91.3 ± 14.2	80.6 ± 9.1
Fats (g/day)	66.5 ± 5.1	59.5 ± 4.8
Saturated fats (g/day)	~6.7 ± 0.5	~6.0 ± 0.6
Unsaturated fats (g/day)	~59.8 ± 0.5	~53.5 ± 0.4
Energy (kcal/day)	3247.6 ± 321.4	2976.3 ± 258.2

Subsequent interviews were conducted by the nutritionist to verify and complete the dietary logs, and dietary monitoring was carried out regularly throughout the duration of the study, which spanned both the pre‐season and season phases, to ensure consistency and adherence to the prescribed dietary guidelines.

The ratio of total unsaturated fat intake was approximately 55% monounsaturated fats and 45% polyunsaturated fats, reflecting a Mediterranean‐style sports diet.

### 2.5 Anthropometric evaluation

All the procedures were conducted in the morning (7:30 a.m.), always before each workout.

Height was measured with a Seca stadiometer to the nearest 0.1 centimeter (cm) with participants barefoot, standing in the upright position with the head in the Frankfort plane. Mass was measured to the nearest 0.1 kilogram (kg) with an electronic Omron balance with the subject wearing minimal clothing.

The percentage of body fat was estimated, following the measurement of three skin folds (chest, abdomen, and quadriceps) with a GIMA mechanical skinfold meter, using the formula developed by Jackson–Pollock (Nevill et al., 2008). The percentage of fat‐free mass was measured using a bioimpedance analyzer (BIA‐AKERN EFG device) (Gatteschi et al., 2011).

All measurements were conducted by the same trained and experienced researcher throughout each phase of the study to ensure methodological consistency and minimize the risk of measurement bias.

### 2.6 Blood parameters

Venous blood samples were taken following fasting in the early morning (8.00 am) following a day off. Blood (10 mL) was collected in vacutainer tubes, using an anticoagulant, and centrifuged at 3000 × g for 20 min to get plasma samples, kept at −20°C until analysis. Serum collected was used to analyze total, HDL, and LDL cholesterol, triglycerides, and glucose. Analysis of the biomarkers mentioned was completed using an automated biochemical analyzer (COBAS c111, Roche, Basel, Switzerland).

### 2.7 Statistical analysis

Results obtained were stored in Microsoft Office Excel 2016 and statistically analyzed by GraphPad PRISM 5 software (GraphPad Software). All variables used in this study were checked for the normality of distribution before the analyses (Kolmogorov–Smirnov tests). To assess changes in anthropometric and lipid parameters (total cholesterol, LDL, HDL, and triglycerides) across the different time points (T0, T1, T2, T3), a Repeated Measures ANOVA (RM‐ANOVA) was performed, considering Timepoint as the within‐subject factor. Mauchly's test of sphericity was used to verify whether the assumption of sphericity was met. For pairwise post‐hoc comparisons between time points, a Bonferroni‐adjusted analysis was conducted to control for multiple testing and reduce the risk of Type I error. A significance level of *p* < 0.05 was adopted. All data were expressed as mean ± standard deviation.

## 3 RESULTS

### 3.1 Anthropometric characteristics of soccer players

**FIGURE 2:**
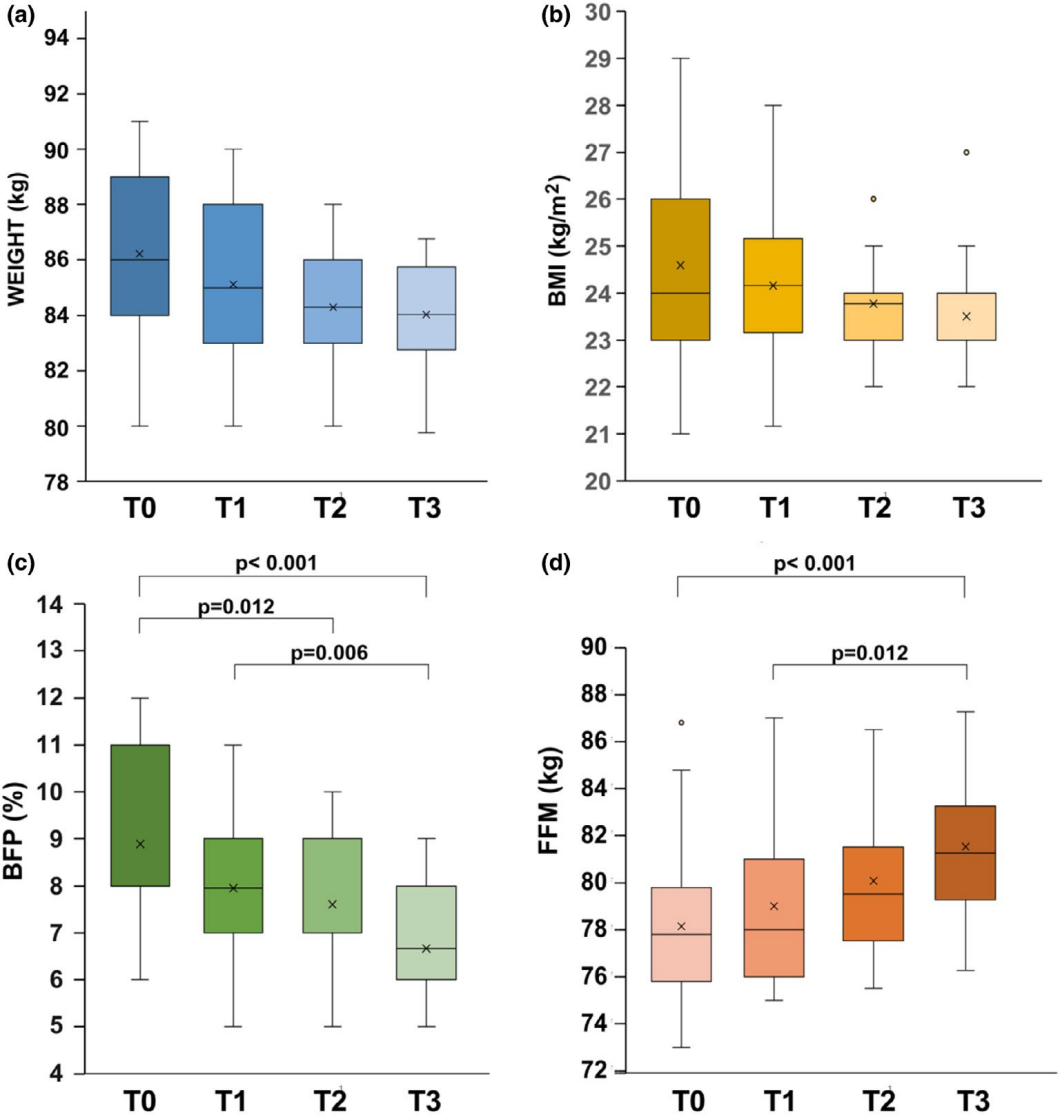
Weight (a), BMI (b), BFP (c), and FFM (d) of soccer players at four time points: Before the start of the training period (T0), immediately after the 4‐week training phase (T1), at mid‐season (T2, 23 weeks after T0), and at the end of the season (T3, 43 weeks after T0). In this representation, the central box covers the middle 50% of the data values, between the upper and lower quartiles. The bars extend out to the extremes, while the central line is at the median. Those values that are beyond 1.5 times the interquartile range beyond the central box are plotted as individual points. *p* is based on post‐hoc comparisons between time points, with Bonferroni adjustment to control for multiple testing.

The anthropometric characteristics of the soccer players weight, body mass index, percentage of body fat (BFP) and fat‐free mass (FFM) were evaluated at the four different time points. During the competitive season, there were significant variations in body fat (BFP, *p* < 0.001 by RM‐ANOVA) and fat‐free mass (FFM, *p* < 0.001 by RM‐ANOVA) as shown in Figure 2. Conversely, weight and BMI did not show any significant variations (*p* > 0.05 by RM‐ANOVA for both).

### 3.2 Cholesterol, triglyceride, and lipoprotein parameters during soccer season

**FIGURE 3:**
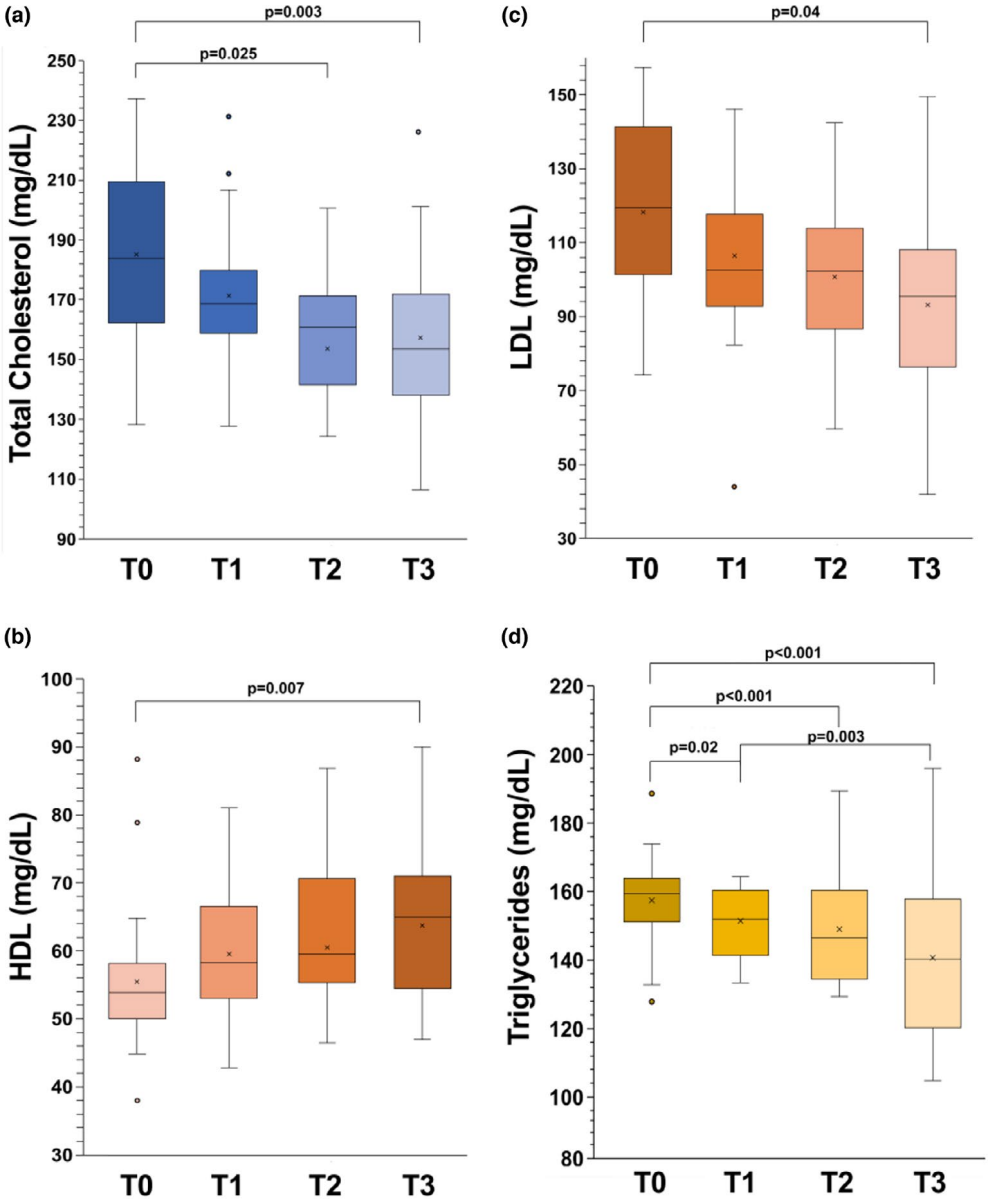
The effects of soccer game season at four time points on serum lipid profile on serum total cholesterol (a), LDL (b), HDL (c), and Triglyceride concentration (d) in soccer players. In this representation, the central box covers the middle 50% of the data values, between the upper and lower quartiles. The bars extend out to the extremes, while the central line is at the median. *p* based on post‐hoc comparisons between time points, with Bonferroni adjustment to control for multiple testing.

Figure 3 shows the lipid profile trends. The plasma levels of total cholesterol (TC) and LDL cholesterol significantly decreased (TC: *p* = 0.003 by RM‐ANOVA and LDL: *p* = 0.033 by RM‐ANOVA), whilst HDL cholesterol concentrations in response to training significantly increased (HDL, *p* = 0.013 by RM‐ANOVA). The triglyceride concentrations were significantly higher before the season than afterwards (*p* < 0.001 by RM‐ANOVA).

**FIGURE 4:**
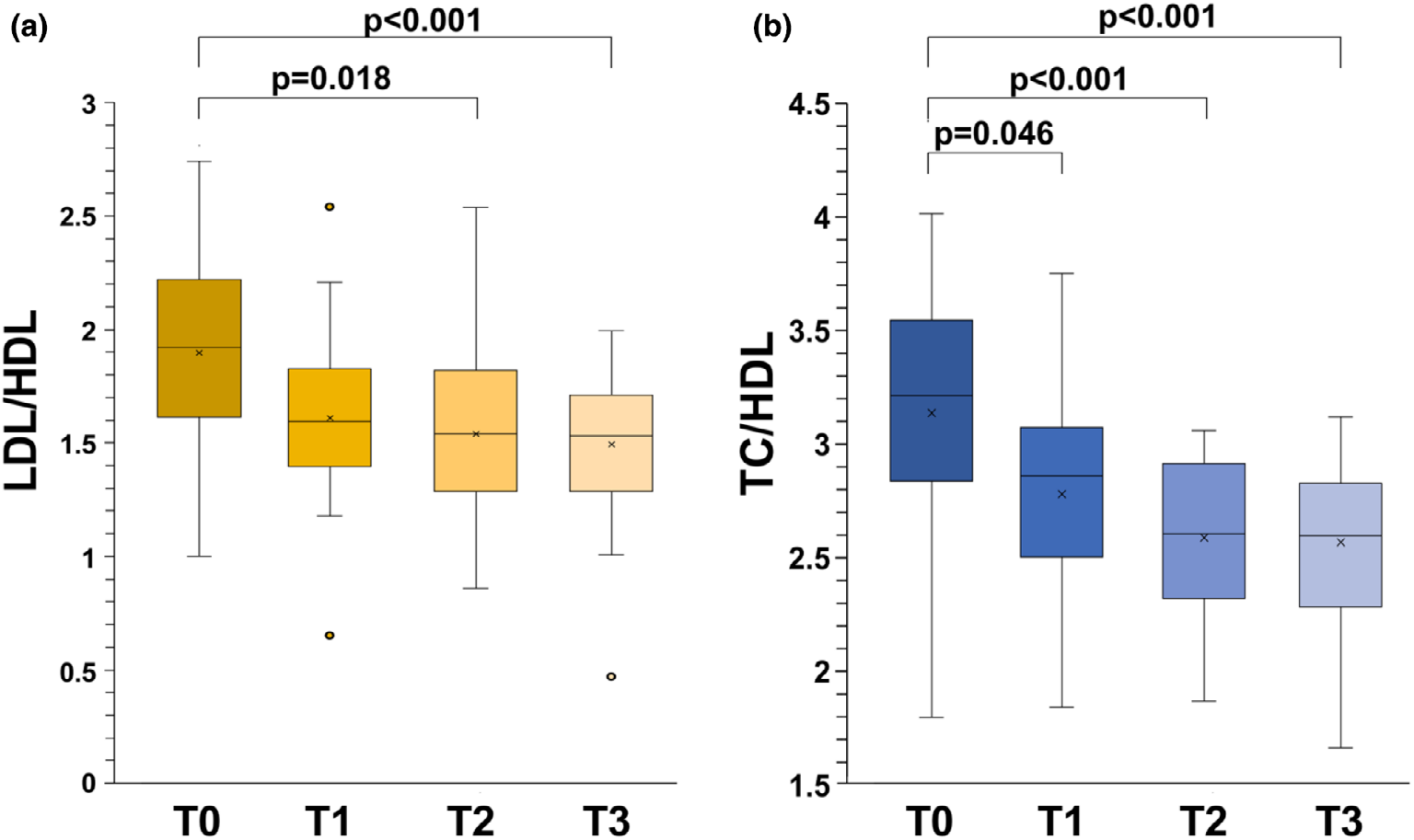
The effects of soccer game season on LDL/HDL (a) and total cholesterol/HDL (b) ratio in soccer players at four time points. In this representation, the central box covers the middle 50% of the data values, between the upper and lower quartiles. The bars extend out to the extremes, while the central line is at the median p based on post‐hoc comparisons between time points, with Bonferroni adjustment to control for multiple testing.

Decrement of LDL/HDL and total cholesterol/HDL ratios during the season were both significant (LDL/HDL: *p* = 0.004 by RM‐ANOVA and TC/HDL: *p* < 0.001 by RM‐ANOVA) and are shown in Figure 4.

## 4 DISCUSSION

A high prevalence of cardiovascular disease risk factors, such as physical inactivity, obesity, and unhealthy diet, has been observed among young adults living in developed countries (Andersson & Vasan, 2018). Consistently, many studies suggest that cardiovascular disease is less common in physically active individuals; this may be due to better endothelial function, reduced inflammation, decreased plasma triglycerides and LDL cholesterol, and increased HDL cholesterol (Pinckard et al., 2019). Furthermore, an increased risk of cardiovascular disease and death has also been shown to be associated with an increase in body mass index (BMI) (Powell‐Wiley et al., 2021).

Among professional soccer players, a lower BMI is associated with a more favorable lipid‐lipoprotein profile (Garry & McShane, 2001). There are also studies reporting changes in anthropometric characteristics and body composition during soccer training periods and their significant impact on player performance (Leão et al., 2022; Sebastiá‐Rico et al., 2023). During the season, we observed a constant decrease in fat mass percentage associated with an increase in lean mass, thus confirming the significant decrease in adipose tissue content during the training period reported by other studies (McEwan et al., 2020; Owen et al., 2018). Our results contrast with those reported by Oliver et al. (2015), who showed an increase in BMI in soccer players during the soccer season without any effect on plasma lipids and cardiovascular disease risk (Oliver et al., 2015). This discrepancy may be partially explained by the different physiological demands of the sports involved. Soccer requires continuous aerobic effort combined with intermittent high‐intensity actions, which tend to promote favorable metabolic and cardiovascular adaptations. In contrast, American football is characterized by short anaerobic bursts followed by longer recovery periods, which may not elicit the same effects on body composition or lipid profile (Bangsbo, 2007; Hoffman, 2008).

Many studies have shown that aerobic and resistance training reduce dyslipidemia (Tesema et al., 2019; Wang & Xu, 2017). However, different exercise interventions, experimental protocols, and participant characteristics in longitudinal studies make it very difficult to quantify the dose of exercise needed to change lipid and lipoprotein levels. Furthermore, HDL‐C and TG are more sensitive to exercise than others (Grässler et al., 2021; Steinmetz et al., 2001). It should be noted that dose–response relationships between exercise volume and changes in blood lipids suggest that even low training volumes may benefit the plasma lipid profile, although effects may not be observable if certain exercise thresholds are not reached (Durstine et al., 2001). Previous studies have evaluated the effects of a soccer match on the plasma lipid profile with conflicting results. Sotiropoulos et al. (2008) demonstrated that HDL levels increased, while TG and total cholesterol decreased after the match. Conversely, Rahnama et al. (2009) showed that soccer matches do not have particularly favorable effects on lipid profiles, but lower levels of LDL, cholesterol, and TG and higher levels of HDL are measured in soccer players, thus suggesting a beneficial effect of regular training on lipid profiles. In light of these results, we can hypothesize that long‐lasting intermittent exercise occurring throughout the soccer season may modify the antiatherogenic lipid profile, probably due to high aerobic energy expenditure. Indeed, our results demonstrate that HDL concentration significantly increased during the soccer season, whilst LDL levels decreased. Given the antiatherogenic role of HDL‐C, its increase is an important result, as is the ratio between TC and HDL‐C in the risk and progression of CHD (Casula et al., 2021). LDL transports cholesterol into peripheral tissue cells where it can be oxidized, thus reaching excessive amounts that accumulate within the walls of the arteries (Franczyk et al., 2023). On the contrary, HDL is important in the removal of cholesterol from peripheral cells, and also from atheroma of the arterial wall to the liver (reverse cholesterol transport); thus, cholesterol contained within HDL is sometimes labeled as “good”. Finally, HDL‐C blood concentration above 60 mg/dL seems to have a protective effect against atherosclerosis and is related to the decrease in the incidence of CHD (Albarrati et al., 2018).

There is conflicting data on the effect of exercise on HDL cholesterol, mainly depending on the type of sport practiced. For example, aerobic exercise performed for 12–24 weeks improves HDL cholesterol by approximately 3.8–15.4 mg/dL from baseline, whereas resistance exercise does not (LeMura et al., 2000). The effect of exercise on HDL and LDL cholesterol concentrations is likely mediated by changes in the concentration and activity of enzymes such as lipoprotein lipase, lecithin/cholesterol acyl transferase, and hepatic triglyceride lipase, implicated in the synthesis, transport, and catabolism of lipoproteins (Muscella, Stefàno, Lunetti, et al., 2020). Exercise‐induced lipid utilization is likely influenced by adipose tissue and intramuscular triglyceride lipolysis, fatty acid transport to exercised muscle, regulation of transmembrane fatty acid transport in muscle cells, and mitochondrial metabolism (Wolfe, 1998). Our long‐term intervention results are consistent with previous studies showing decreases in total cholesterol (TC), triglycerides (TG), and low‐density lipoprotein cholesterol (LDL‐C) and increases in high‐density lipoprotein cholesterol (HDL‐C) in basketball and soccer players (Afşin et al., 2021; Apostolidis et al., 2014; Krustrup et al., 2009; Papapanagiotou et al., 2015). Furthermore, soccer players had baseline HDL cholesterol values that were 18% higher and a 15% lower TC/HDL ratio than non‐athletes, presumably because they had significantly higher energy expenditure than sedentary individuals (Amanatidis et al., 2006). However, LDL levels increased significantly after the soccer match, although they were still within the recommended range (<130 mg/dL) (Sotiropoulos et al., 2008).

This may be because the increased free radical production associated with aerobic exercise can influence the oxidative activity status of circulating LDL particles immediately after the match (Sánchez‐Quesada et al., 1995). Since the TC/HDL‐C ratio has been proposed as another risk indicator for CHD (Quispe et al., 2020), our results show that long periods of soccer training can cause significant changes in this ratio, consistent with previous studies performed on 12‐week intervention periods of soccer training that caused an LDL and LDL/HDL decrement in untrained men (Bangsbo et al., 2015; Krustrup et al., 2009). On the other hand, improvements in these lipid variables appear to be associated with a reduction in body mass and body fat percentage (Lemieux et al., 2001; Zhang et al., 2023). Our findings are consistent with those previously observed when the recreational soccer intervention period was longer than 12 weeks, causing a significant decrement in lean body mass and body fat percentage (Bangsbo et al., 2015; Knoepfli‐Lenzin et al., 2010).

Conflicting results have been reported regarding TG levels assayed in soccer players, with studies reporting lower values (Feairheller et al., 2016; Hurst et al., 2012; Selden et al., 2009) and others higher values than in control groups (Allen et al., 2010; Dobrosielski et al., 2010; Haskins et al., 2011). For example, baseball players have lower TG values than soccer players (Helzberg et al., 2010); however, and consistently with previously reported studies, significant decreases occur during the season (Afşin et al., 2021; Apostolidis et al., 2014; Krustrup et al., 2009; Papapanagiotou et al., 2015). Although exercise is known to affect intramuscular TG lipolysis (Muscella, Stefàno, Lunetti, et al., 2020), sedentary individuals show no change in TG levels after a single session of exercise (Wang & Xu, 2017). Thus, a longer period of intervention would appear to be necessary to produce significant effects on the lipid profile. Furthermore, the aforementioned variations appear to be related to the training program and variations in VO_2_max (Heath et al., 1983). Soccer players use different energy systems based on activity intensity. During high‐intensity efforts– like matches, sprints, or drills–they primarily rely on carbohydrates. At lower intensities, especially between 45% and 65% of VO_2_max (e.g., recovery runs or aerobic conditioning), the body mainly uses fatty acids (Burke et al., 2004; Sidossis et al., 1998). Since players alternate between high and low intensities throughout the week, their muscles shift between carbohydrate and fat use. This may help explain the variability in fat metabolism and lipid markers seen in studies on soccer players.

It is important to contextualize the energy systems involved. Although elite soccer players predominantly rely on carbohydrate oxidation during high‐intensity match play and training (via both anaerobic and aerobic glycolysis), their training regimen also includes lower‐intensity sessions, such as recovery and aerobic conditioning. During these phases, fatty acids derived from adipose tissue, muscle lipid droplets, and diet serve as the main energy sources, especially at exercise intensities between 45% and 65% of VO_2_max (Burke et al., 2004; Sidossis et al., 1998). Therefore, at low to moderate intensity and during prolonged efforts, skeletal muscle energy needs are met primarily by fatty acid oxidation, with a smaller contribution from glucose oxidation. This dual‐energy system activation across the training microcycle may partly explain the variability observed in lipid‐related outcomes.

Our results provide valuable insights into the impact of a competitive soccer season on the lipid profiles of elite players, a population generally characterized by optimal cardiovascular health. We demonstrated that plasma concentrations of total cholesterol (TC) and LDL cholesterol decreased while HDL cholesterol increased during the competitive season; thus, a decrease in LDL/HDL and TC/HDL ratios was also observed. Lipids are considered an important source of energy during exercise, and the regulation of their metabolism is a complex task. During prolonged exercise, adipose tissue and intramuscular lipolysis are regulated by both contraction and hormonal mechanisms. However, the mechanisms for increased lipid metabolism remain to be fully understood, as there are many functional and structural steps during the mobilization, transport, and oxidation of fatty acids in working muscle.

A major limitation of our study is the lack of direct data on substrate utilization. Although we focused on blood parameters and body composition changes, this limits the understanding of how the training program specifically influences substrate metabolism. Future studies should aim to include such techniques to provide a more comprehensive picture of energy metabolism during both training sessions and recovery periods. Another limitation of this study is the lack of detailed data on physical activity levels during the off‐season period. Because this period typically involves reduced and unstructured activity, it may have influenced basal metabolic state and subsequent response to training. Future studies should include objective monitoring of off‐season activity (e.g., via GPS tracking or activity logs) to better contextualize physiological adaptations during the season.

While our study was designed primarily for elite soccer players, we acknowledge that the insights gained could have broader implications. The patterns of training and physical activity observed in this population may offer valuable knowledge on the potential benefits of regular physical exercise for general health. Well‐designed, structured exercise programs—scaled to individual fitness levels and incorporating both endurance and resistance elements—could likewise support improvements in lipid profiles and reductions in cardiovascular risk factors in the broader population. However, it is important to note that future research would be needed to directly investigate how these findings might translate to non‐athletic populations, taking into account differences in baseline health status, activity levels, and training adaptation.

## CONFLICT OF INTEREST STATEMENT

The authors declare that they have no known competing financial interests or personal relationships that could have appeared to influence the work reported in this paper.

## ETHICS STATEMENT

This study was approved by the Institutional Review Board (I.R.B.) of the Department of Biological and Environmental Science and Technologies (Di.S.Te.B.A.) (protocol code N‐1/2021 and 7 January 2021).

## INFORMED CONSENT STATEMENT

Informed consent was obtained from all subjects involved in the study.

## Supporting information


Data S1.

## Data Availability

Data will be made available on request.
