# Supplementary material for: Effects of a competitive season on the plasma lipid profile of soccer players
Source: Physiol Rep. 2025 May 27;13(10):e70382. doi: 10.14814/phy2.70382 (PMC12106951; doi:10.14814/phy2.70382)
Supplement: Supplementary file 1 — Data S1. [file PHY2-13-e70382-s001.docx]

**Table. S1 Team** training program in period of athletic preparation (pre-season).

| **Day** | **Session Type** | **Dose (Km, AU)** | **Intensity (%HRmax)** | **Time (min)** |
| --- | --- | --- | --- | --- |
| **MD** (Match Day) | Official Match | 9–12 km ~1000–1200 AU | 85–95% | 90–100 |
| **MD+1** (Recovery) | Recovery / Regeneration | 2–4 km, ~100–200 AU | 50–60% | 30–45 |
| **MD+2** (Light Training) | Rest or Light Training | 0–3 km, ~0–150 AU | 40–60% | 0–60 |
| **MD-4** (Tactical) | Tactical + Physical | 5–7 km, ~300–500 AU | 70–85% | 60–75 |
| **MD-3** (High Intensity) | High Intensity Day | 6–8 km, ~500–700 AU | 80–90% | 60–75 |
| **MD-2** (Tactical) | Tactical / Set Pieces | 4–6 km, ~250–400 AU | 65–80% | 45–60 |
| **MD-1** (Activation) | Activation, Flexibility | 2–4 km, ~150–250 AU | 60–70% | 30–45 |

Km, total distance; AU, (sRPE x min)

**Table S2** Team training program in season

| **Day** | **Session Type** | **Dose (Km, AU)** | **Intensity (%HRmax)** | **Time (min)** |
| --- | --- | --- | --- | --- |
| **MD-4** (Tactical + Physical) | Aerobic + Strength (core stability, gym) | 5–7 km, ~300–500 AU | 70–85% | 60–75 |
| **MD-3** (High Intensity Day) | Speed, Aerobic, Isotonic Gym | 6–8 km, ~500–700 AU | 80–90% | 60–75 |
| **MD-2** (Tactical / Set Pieces) | Technical-Tactical (low intensity) | 4–6 km, ~250–400 AU | 65–80% | 45–60 |
| **MD-1** (Pre-match Activation) | Activation, Flexibility | 2–4 km, ~150–250 AU | 60–70% | 30–45 |
| **MD+1** (Recovery/ Regeneration) | Recovery (low intensity) | 2–4 km, ~100–200 AU | 50–60% | 30–45 |
| **MD+2** (Rest or Light Training) | Active Recovery / Light Training | 0–3 km, ~0–150 AU | 40–60% | 0–60 |

Km, total distance; AU, (sRPE x min)
